# Phenotypic diversity and shared genomic determinants among isolates causing a large incidence of disseminated gonococcal infections in Canada

**DOI:** 10.1128/msphere.00051-26

**Published:** 2026-07-20

**Authors:** Gursonika Binepal, Emil Jurga, Duncan Carruthers-Lay, Sören Krüger, Sandra Zittermann, Jessica Minion, Mathew Diggle, David C. Alexander, Irene Martin, Vanessa Allen, John Parkinson, Scott D. Gray-Owen

**Affiliations:** 1 Department of Molecular Genetics, University of Toronto 7938, Toronto, Ontario, Canada; 2 Program in Molecular Medicine, Hospital for Sick Children Research Institute 483367, Toronto, Canada; 3 Department of Respiratory Medicine, Leipzig University 9180, Leipzig, Germany; 4 Department of Intensive Care Medicine, University of Aachen, Aachen, Germany; 5 Public Health Ontario 153300, Toronto, Ontario, Canada; 6 Department of Pathology and Laboratory Medicine, University of Saskatchewan 7235, Saskatoon, Canada; 7 Roy Romanow Provincial Laboratory (formerly Saskatchewan Disease Control Laboratory), Regina, Saskatchewan, Canada; 8 Alberta Precision Laboratories-Public Health Laboratory (ProvLab) 113196 , Edmonton, Alberta, Canada; 9 Cadham Provincial Laboratory, Diagnostic Services, Shared Health, Winnipeg, Manitoba, Canada; 10 National Microbiology Laboratory, Public Health Agency of Canada, Canadian Science Centre for Human and Animal Health 60415, Winnipeg, Canada; 11 Department of Biochemistry, University of Toronto 7938, Toronto, Ontario, Canada; 12 Centre for Global Child Health, Hospital for Sick Children, Toronto, Ontario, Canada; US Food and Drug Administration, Rockville, Maryland, USA

**Keywords:** *Neisseria gonorrhoeae*, disseminated gonococcal infection (DGI), gonorrhea, sexually transmitted infection (STI), phenotypic analysis, genomic analysis

## Abstract

The incidence of disseminated gonococcal infection (DGI) has remained low since the advent of antibiotics; however, a recent surge in DGI has inexplicably emerged during the past decade. In an effort to understand whether *Neisseria gonorrhoeae* that cause disseminated disease can be differentiated from non-invasive strains, we have performed a phenotypic and genotypic analysis on a selection of isolates obtained from invasive and uncomplicated infections in Canada. The increase in DGI was initially associated with a single antibiotic-susceptible gonococcal lineage, but later cases have been caused by diverse strains. Phenotypic analysis of a matched subset of 20 isolates obtained since 2013 found that these varied in their capacity to aggregate in suspension and in their association with serum complement proteins; however, these interactions did not discriminate between the invasive and mucosal isolates. Sequence typing of 360 Canadian isolates revealed that two variant porBa alleles are significantly associated with the DGI strains, one of these being present throughout the past decade, whereas the other emerged more recently. A PopNet-based population dynamics analysis, which instead establishes relationships based upon variance among discrete chromosomal segments, revealed that DGI isolates were restricted to distinct subpopulations within their phylogenetic distribution, implying a genetically linked potential to cause invasive disease. Consistent with this, a large number of genetic determinants are enriched in the DGI strains, making these enticing candidates to explain the increased capacity of *N. gonorrhoeae* to cause systemic infection and/or reduce the presentation of clinical symptoms from localized infection so that it remains untreated.

**IMPORTANCE:**

The re-emergence of disseminated gonococcal infections (DGIs) has raised concern that the virulence of some strains has increased due to their acquisition of a new capacity to remain asymptomatic during uncomplicated mucosal infection and/or to invade the bloodstream and persist upon establishing colonization at distal tissue sites. The molecular epidemiology of a very large incidence of DGI in Canada, which began from a single strain but then diversified with time, provided an opportunity to understand whether the invasive isolates were either phenotypically or genotypically related. While classical phenotypic analysis did not discriminate between mucosal and invasive isolates, a population dynamics-based approach established that the DGI isolates are present within distinct subpopulations of *Neisseria gonorrhoeae* and reveals genetic determinants that are linked to this phenotype. This suggests that the DGI phenotype emerged multiple times independently and/or the genetic features responsible have been transmitted among the populations.

## INTRODUCTION

*Neisseria gonorrhoeae* (*Ngo*) causes over 82 million infections per year globally (1). This high burden of gonococcal infection and its progressive acquisition of resistance to antibiotics has created an increased urgency for understanding its epidemiology and developing new anti-gonococcal therapeutics (1). Symptomatic infections typically present with localized urogenital manifestations, including profuse urethral or cervical discharge, though asymptomatic colonization of these or other mucosal sites is common. The highest burden of disease occurs in untreated women, where *Ngo* can spread into the upper genital tract to cause inflammation and tissue scarring, with sequelae including chronic pain, ectopic pregnancies, and infertility (2). In rare cases, disseminated gonococcal infections (DGIs) can arise in both men and women, with presentations including migratory polyarthralgia, arthritis, tenosynovitis, endocarditis, and/or dermatitis (3, 4). DGI has been reported to occur in 0.5%–3% of those infected (3, 5, 6), though other studies suggest that rates are now typically below this (4), presumably due to the success of treatment in preventing the disease. Recently, unexplained clusters of high DGI incidence have been reported in Manitoba, Michigan, Georgia, and California (7–10). All gonococcal infections have increased, but the proportion of cases that manifest as DGI has been significantly higher in these regions without obvious increases in other areas. Also notable is that DGI has historically been higher in women, while these more recent emergences appear to affect men and women equally, suggesting some unique characteristics of these recent cases.

Gonococcal isolates from disseminated sites have long been known to resist the bactericidal activity of normal human serum (HS) (11). This has been attributed, at least in part, to the unique ability of gonococcal strains to variably bind complement regulatory factors that normally function to protect human cells from complement-dependent killing, including factor H (FH), C4b-binding protein (C4BP), and vitronectin. Gonococcal sialylation of its lipo-oligosaccharide (LOS), neisserial surface protein A (NspA), and the porin B (PorB) variants can each, synergistically, promote FH binding to the bacterial surface, which confers resistance to the alternative (immunoglobulin-independent) complement cascade (reviewed in reference 12). Certain variants of PorB also bind human C4BP, which inhibits immunoglobulin-dependent (classical) complement killing. C4BP binding is most commonly apparent with expression of a *porB1a* allele. This is not a strict association given that the interaction is evident in 80% of strains expressing PorB1a and 20% of those expressing the closely related but antigenically distinct PorB1b (12), yet the relationship is consistent with the fact that invasive isolates tend to express PorB1a (12, 13). The diversity of mechanisms by which the gonococci can decorate their surface with complement regulatory factors to resist the bactericidal activity of serum, and the absence of any other factors known to increase the propensity of *Ngo* to cause systemic infection, makes it difficult to understand why individual strains may be more or less likely to cause DGI.

Given the genetic diversity of *Ngo*, it is notable that phylogenetic analysis of the isolates recovered from the DGI cases in Michigan (7) and Manitoba (8) indicates that they are highly related, with the latter being largely associated with *Ngo* having closely related multi-antigen sequence types (NG-MAST), while those isolated in Georgia were more genetically diverse (10). A marked increase in DGI has also been apparent in Ontario (14), which abuts both Manitoba and Michigan, first emerging in 2014 and then remaining at elevated rates since that time. Genomic sequence analysis by Public Health Ontario (PHO) indicates that the early strains were of the NG-MAST that has been associated with most DGI in Manitoba (type 11508), suggesting that they are epidemiologically linked; however, later strains have diverged from this. Herein, we have taken advantage of the routine collection of gonococcal isolates by public health agencies in Canada to characterize the relative capacity of disseminated and non-disseminated (mucosal) isolates from 2013 through 2019 to bind complement factors and resist the bactericidal activity of human blood, and to search for genetic determinants that are uniquely shared by the diverse isolates recovered.

## RESULTS

### Serum resistance of low-passage *Neisseria gonorrhoeae* isolates

**Fig 1:**
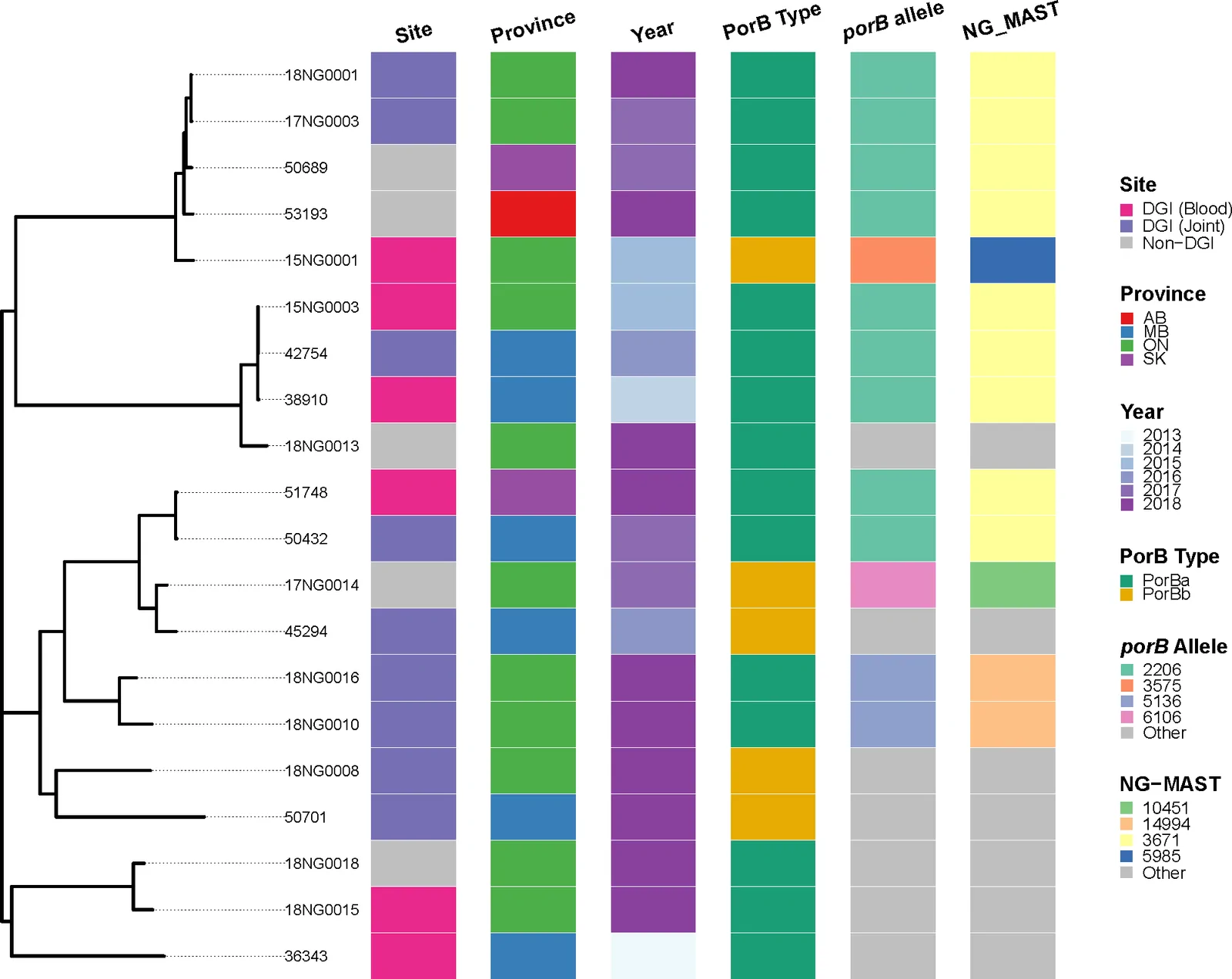
Characteristics of *N. gonorrhoeae* clinical isolates. Maximum likelihood tree of 20 isolates selected for phenotypic analysis, constructed from a concatenated set of SNPs generated from alignment to the FA1090 reference genome. Only branches with bootstrap support >0.8 are shown. Columns (outside-in) represent NG-MAST serotypes, PorB allelic variant, PorB type, year of collection, province, and DGI phenotype.

DGIs are typically considered to emerge from asymptomatic and/or untreated genital infections. The propensity for certain strains to be associated with invasive infections suggests that they possess some capacity that is not shared by strains that remain localized to the genital or other mucosal surfaces. To phenotypically distinguish isolates that do or do not have the potential to cause invasive disease, we selected a subset consisting of 20 clinical isolates obtained between 2013 and 2019, including 15 isolated from invasive infection and 5 isolates with similar NG-MAST type but from uncomplicated genital infection (Table S1A). Of these, 11 were from Ontario, 6 from Manitoba, 2 from Saskatchewan, and 1 from Alberta. Both types of porin B, the most abundant antigen in the outer membrane and a key element of molecular typing schemes, are represented, with 15 PorB1a (5 DGI-blood, 6 DGI-joint, and 4 non-DGI) and 5 PorB1b (1 DGI-blood, 3 DGI-joint, and 1 non-DGI) isolates. These attributes are depicted relative to their phylogenetic relationship, based upon concatenated single nucleotide polymorphisms (SNPs) generated after alignment with the FA1090 genome, in Fig. 1.

**Fig 2:**
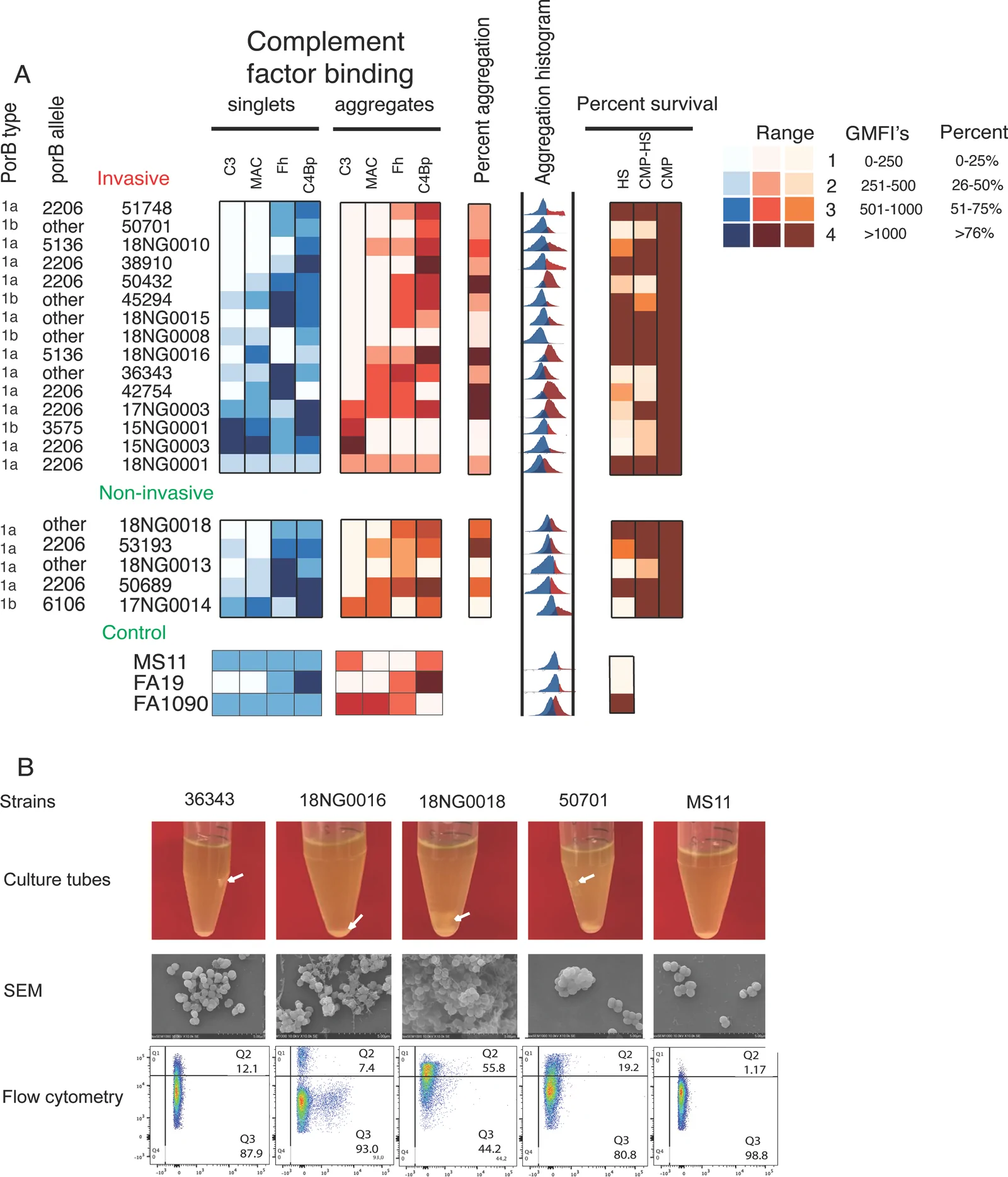
Serum resistance and complement binding assays. (**A**) PorB type and porB alleles are shown for each strain. Binding of indicated (C3, C5b-9 complex of the membrane attack complex [MAC], FH, and C4BP) complement factors to bacterial singlets (blue) and aggregates (red) after treatment with 60% HS are shown as heatmaps. The percentage of singlets versus aggregated bacteria observed in normal (no serum) media (percent aggregation) is indicated beside flow cytometry plots (aggregation histogram) illustrating the shift in population from singlets (blue) to aggregates (red). Serum resistance was calculated to obtain percent CFU recovery (percent resistance) and then plotted as a heatmap based upon quartiles indicated in the legend. The effect of culturing in CMP-NANA (CMP) and/or HS on the percent survival of indicated strains is shown. Values represent normalized fluorescent values (geometric mean fluorescence intensity: 0–250, 251–500, 501–1,000, >1,000). (**B**) Aggregation of gonococci in normal growth media. Representative gonococcal strains were grown in BHI supplemented with IsoVitalex at 37°C shaking at 180 rpm for 6–8 h. Images depict differential bacterial settling (clumping) in the culture tubes. Scanning electron micrographs of the bacterial suspensions showing clumping. Flow cytometry dot plots gated to illustrate singlets (bottom right quadrant) and larger aggregates indicative of clumping (upper right quadrant).

Given that resistance to the complement-mediated killing is presumed to be a prerequisite for systemic infection, we next assessed the susceptibility of these isolates to human serum. We first tested the serum concentration (10% to 90% HS) with five randomly selected strains (15NG0003, 17NG0014, 18NG0016, 36343, and 53193) (Fig. S1). Based on colony-forming count data, all the test strains survived below a concentration of 60% HS, while some were unrecoverable above this, so this was selected to differentiate serum sensitivity between the isolates. If the CFU recovery after this treatment was below 50%, they were considered serum sensitive; strains with CFU recovery from 50%–75% were considered partially resistant, and those with over 75% CFU recovery were considered serum-resistant strains. We applied this to the full set of 20 clinical isolates, comparing the susceptibility of each strain in the presence or absence of 5′-cytidinemonophospho-*N*-acetylneuraminic acid (CMP-NANA) prior to 60% HS exposure because some strains can use this to sialylate their LOS and thereby increase their survival (12). In each case, we quantified the percent of bacteria recoverable (Fig. 2).

The recovery of strains was not reduced by pre-culturing with CMP-NANA (CMP, Fig. 2A). For isolates from uncomplicated infections, three of five were resistant in human serum, and one more became resistant to HS by pre-culture in CMP-NANA (CMP-HS; total 80% of strains resistant). From DGI, 7 of 15 isolates were serum resistant, and 4 more became resistant with CMP-NANA (11 total, 73% resistant). Considering the PorB types, 9 of 15 PorB1a-expressing isolates were serum resistant, and an additional 4 became resistant after growth in CMP-NANA (13 total, 93% resistant). Two of the 3 DGI isolates that express PorB1b were serum resistant, while the other was sensitive whether or not it was pre-cultured with CMP-NANA (66% resistant). The mucosal isolate that expresses PorB1b was HS sensitive but became resistant after pre-growth with CMP-NANA.

Sawatzky et al*.* found a PorB1a variant encoded by the *porBa-2206* allele to be typically expressed by *Ngo* isolates associated with the recent outbreak of DGI in Manitoba (8). In our subset, 7 of 15 PorB1a isolates carried the *2206* allele (Fig. 1), and 5 of these 7 isolates exhibited high serum resistance. The two non-invasive isolates (53193 and 50689) that carried this allele (Fig. 1B) were also highly serum resistant. The *porB-5136* allele was observed exclusively in two invasive isolates (18NG0010 and 18NG0016) that exhibited high serum resistance, while the allele *porB-3575* was only present in one invasive isolate, 15NG0001. More details on these alleles are under the clonal lineage below.

### Complement factor binding does not differentiate invasive and genital isolates

Considering that *in vitro* serum bactericidal activity (SBA) alone did not reveal how *Ngo* interacts with individual serum components, we next sought to quantify complement protein deposition. In addition to monitoring the two human-derived negative regulators of complement activation, C4BP and factor H, both of which facilitate *Ngo* complement resistance (12), we used bacterial binding to C3 and the presence of a C5b-9 complex-dependent neoepitope as direct measures of opsonization and the membrane attack complex (MAC), respectively. During these analyses, we noted a tendency for some strains to form aggregates (Fig. 2B) in/or when suspended in human serum (Fig. S2B). To consider the effect of this, we have depicted this as an “aggregation histogram,” which differentiates the single-cell (blue) versus aggregate (red) populations, and quantified “percent aggregation” to reflect the propensity of strains to aggregate based upon the frequency of single cells (singlets) versus aggregates (Fig. 2A). Notably, all four strains that displayed the highest aggregation (indicated by darkest shading in percent aggregation) were invasive; however, two of these (50432 and 42754) were serum sensitive in both the absence and presence of CMP-NANA.

Since aggregation would alter surface accessibility to complement factors, we evaluated the binding activities for aggregates and singlets separately (see Materials and Methods). Some differences are apparent among individual strains; there were no significant trends other than that some strains bound higher FH as singlets than as aggregates (Fig. S2C). While individual strains displayed marked variation in their level of binding to C3 and MAC, we observed no distinction between DGI and mucosal isolates, and no clear difference between PorB1a- and PorB1b-expressing strains (Fig. 2A and Fig. S2A, C, and D). Most strains displayed substantial binding to FH and/or C4BP and low or no C3 or C5b-9 binding, consistent with their being highly resistant to complement killing. Four strains were highly serum sensitive (<50% survival in HS), and this was not significantly affected by pre-culturing the bacteria in CMP-NANA. Of these, strain 50701 (DGI, PorB1b) displayed very low binding to C3 and MAC. Based upon this analysis, the increased propensity to cause invasive gonococcal infections is not reflected by any strict correlation with serum resistance or complement factor binding.

### DGI isolates are from diverse clonal lineages

We next took an unbiased approach to consider that an as yet undescribed function might contribute to the DGI phenotype. For this purpose, we took advantage of the availability of genome sequence data collected from 360 isolates during surveillance for the ongoing incidences of DGI in Canada. Of the 360 isolates, 184 were recovered from genital infections, 48 from rectal infections, 25 from eye infections, 20 from the throat, 9 had unconfirmed sites of isolation, and 74 were reported as DGI (Table S1). While most (272 of 360) of these isolates were derived from male patients, the DGI isolates were relatively evenly divided between males (40) and females (32) (Table 1), reflecting that seen recently in these regions.

**TABLE 1:** Summary of isolates used in the study

Parameter	Number of samples		
	Total	Non-DGI	DGI
Year			
2013	1	0	1
2014	9	5	4
2015	135	131	4
2016	80	77	3
2017	53	34	19
2018	67	38	29
2019	15	1	14
Gender			
Male	272	232	40
Female	81	49	32
Unknown	7	5	2
Age			
10–19	26	19	7
20–29	143	126	17
30–39	92	73	19
40–49	64	46	18
50–59	14	10	4
60–69	12	6	6
70–79	2	1	1
Unknown	7	5	2
PorB allele			
porBa	115	60	55
porBb	230	224	6
Unknown	15	2	13
Province			
Ontario	294	254	40
Manitoba	59	27	32
Saskatchewan	5	3	2
Alberta	2	2	0

**Fig 3:**
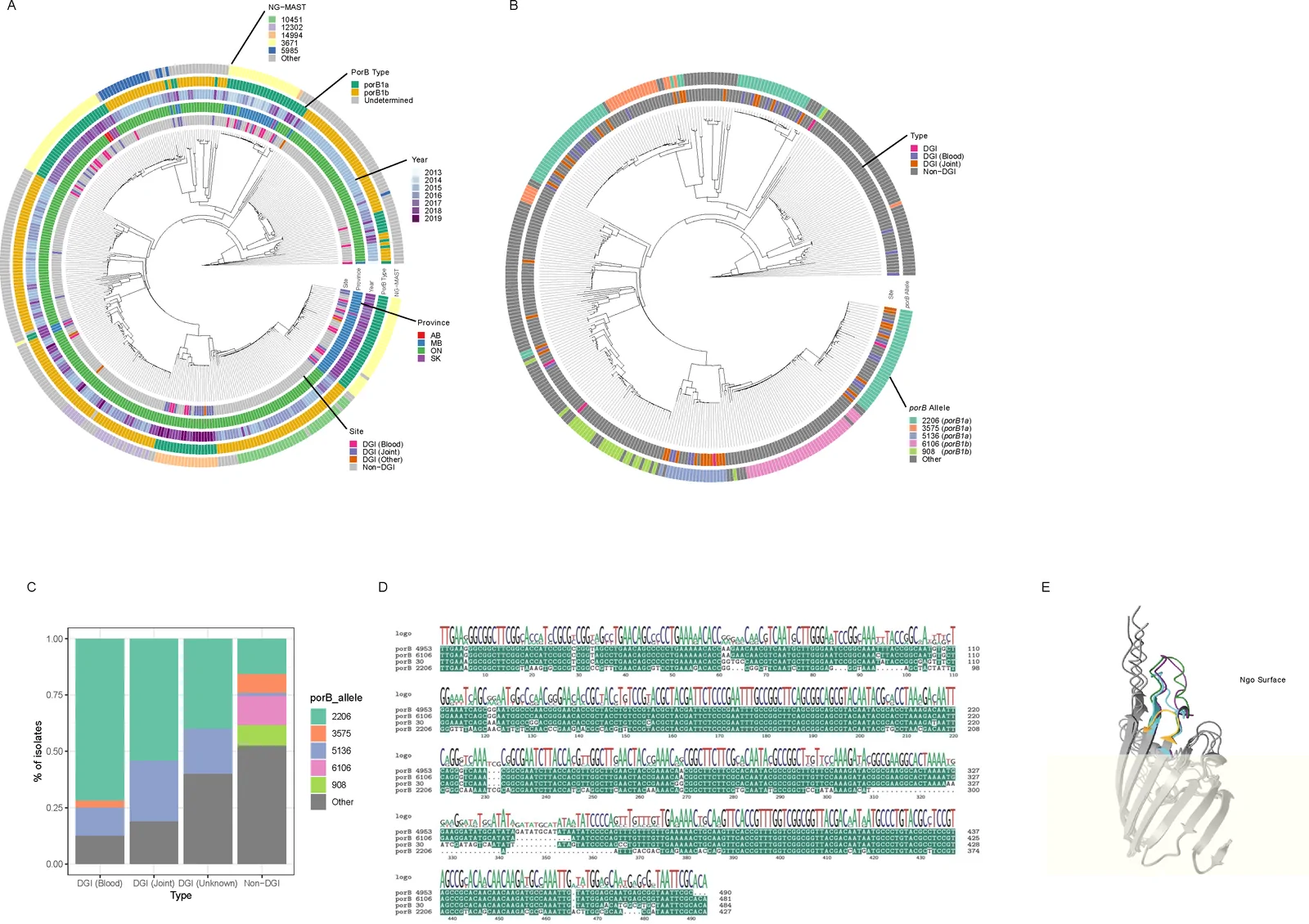
Phylogenetic relationship between 360 *N. gonorrhoeae* isolates. Maximum likelihood tree of 360 isolates constructed from a concatenated set of SNPs generated from alignment of isolate genomes against the FA1090 reference genome. Only branches with bootstrap support >0.8 are shown. (**A**) Outer rings (outside-in) represent NG-MAST serotypes, *porB* allelic variant, year of collection, province, and DGI phenotype. (**B**) Outer rings (outside-in) represent *porB* allele and DGI phenotype. (**C**) Proportion of isolates carrying *porB* alleles associated with DGI phenotypes. Only the five most common *porB* alleles are shown. (**D**) Sequence alignment of four *porB* alleles; 2206 (*porBa)*, 6016 (*porBb*, non-DGI), 30 (*porBb*, DGI-associated), and 4953 (*porBb*, DGI-associated). (**E**) Structural alignment overlaying four *porB* alleles; 2206 (yellow), 6106 (cyan), 30 (green), and 4953 (purple). The variable loop 5 region is colored to highlight allele-specific differences.

To assess the genetic variation of the isolates, genome sequences for each isolate were aligned to that of the reference strain, FA1090, a serum-resistant strain originally isolated from the cervix of a patient with disseminated gonococcal infection (15). Based on concatenated alignments of the single SNPs between these sequences, we constructed a phylogenetic tree based on maximum likelihood (Fig. 3A). While the earliest isolates (2013–2015) tend to be from Manitoba, we otherwise observe little population structure associated with geography, *porB* allele, or NG-MAST, reflecting the inherent genetic diversity of gonococcal populations (16), even within this relatively restricted region. NG-MAST allele typing of the *porB* gene revealed two *porBa* alleles, *2206* (45/90 DGI) and *5136* (15/19 DGI), that are significantly associated with DGI isolates (Fisher’s exact test, *P* < 0.00001). Allele *2206* was found in isolates across Canada (56 from MB, 27 from ON, and 7 from SK/AB), while all isolates with allele 5136 were collected in Ontario. Both alleles were represented in DGI isolates collected from blood and synovial fluid, although more isolates with 5136 were recovered from the latter (10 in synovial vs 4 in blood).

Of the 74 DGI isolates included in this study, 66 possessed the porB1a allele, which has previously been linked to the invasive phenotype. We note that the remaining eight DGI isolates possess the porB1b allele, again suggesting that additional factors must be associated with the ability to cause invasive disease. In total, 13 different *porB* alleles were associated with DGI isolates (Fig. 3A through C). We found five DGI-associated *porB1b* type alleles (3575, 6435, 30, 4953, 6892) and compared their sequences to the most abundant non-DGI-associated *porB1b* allele, 6106, focusing on the variable loop 5 that differentiates *porB* types. Alleles 30, 3575, and 6435 were highly similar, with point mutations observed at positions 330–336. We also observed a 9 base pair insertion at position 337 in allele 4953 (Fig. 3D). Allele 6892 was identical to allele 6106 except for a point mutation (A to G) at position 331 and was therefore not shown. Allele 30 was chosen to represent alleles 30, 3575, and 6435. In considering how the DGI-associated PorB1b proteins compared to DGI-associated PorB1a, we looked at the structural properties of these variants (Fig. 3E), with the gray representing mostly static regions and the variable loop 5 region is colored with *porB1a* alleles *2206* (yellow), non-DGI-associated *porB1b* allele 6106 (blue), and the DGI-associated *porB1b* alleles 30 (green) and *4953* (purple). While the localization of these sequence changes, being situated within the region deleted in *porB1a* alleles, may indicate some alterations in PorB function, it remains unknown whether its binding and/or other properties would be affected.

### PopNet analysis identifies subpopulations associated with the DGI phenotype

**Fig 4:**
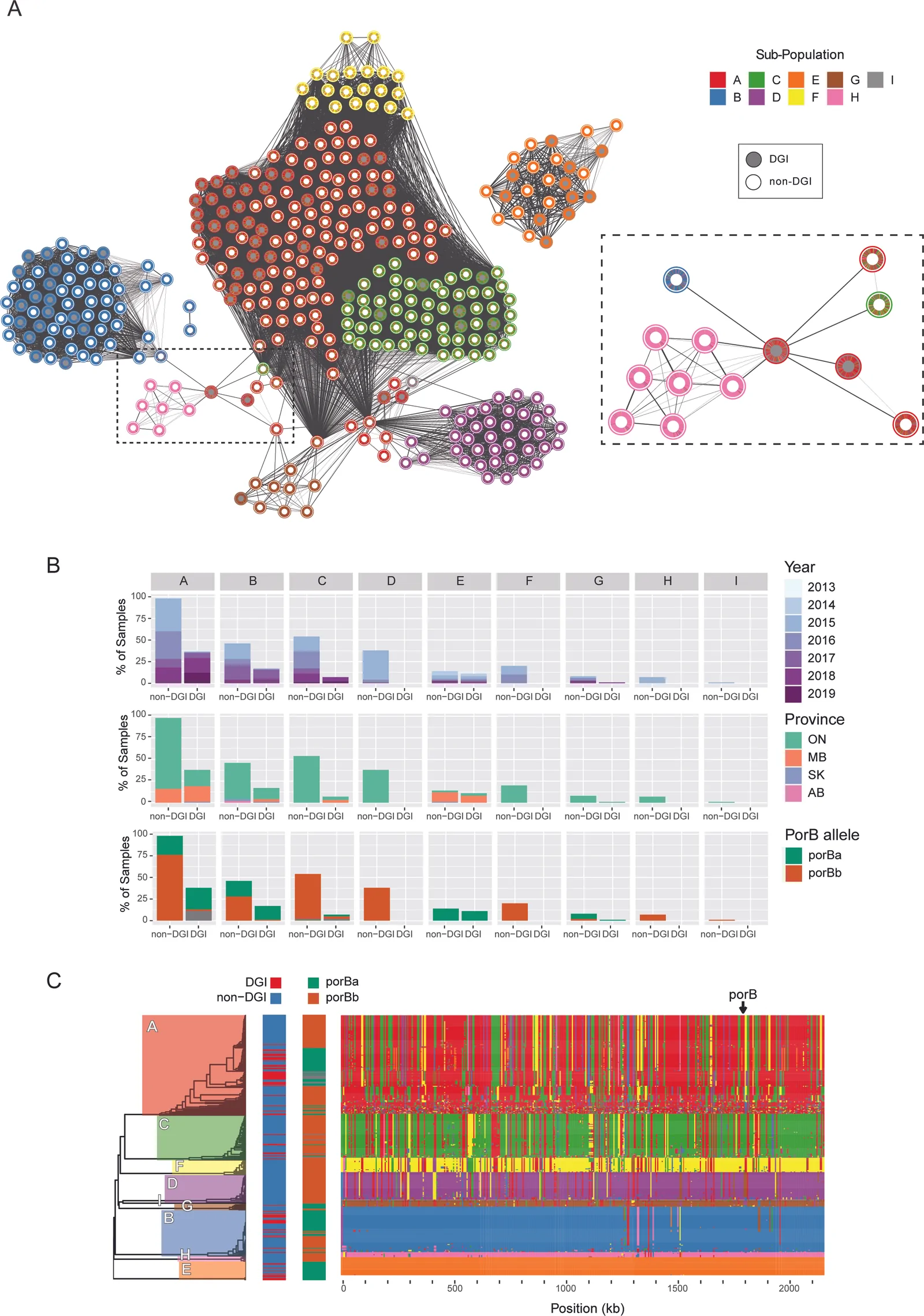
Detailed analysis of subpopulations defined by PopNet. (**A**) PopNet representation of isolates. In this network, nodes represent individual isolates and edge (line) thickness represents the percent similarity, in terms of shared SNPs. Only edges representing >15% similarity (defined as the percentage of segments that co-cluster) were used to construct the network. Nodes (isolates) are represented by annuli where colored segments represent regions of the chromosome which share ancestry across subpopulations. Filled (gray) or open (white) shading within the annuli indicate whether the isolate was associated with DGI or not. Inset, zoom-in of the indicated (dashed line) set of isolates showing how one isolate links between multiple (red, blue, pink, and green) subpopulations, suggesting that the isolate may be a descendant of an ancestral isolate responsible for the emergence of these subpopulations. (**B**) Proportional analysis of the attributes associated with each subpopulation, showing year of collection, province of isolation, and PorB allele type. Percent of samples shown is calculated relative to the largest group of strains (subpopulation A non-DGI). (**C**) Linear chromosome painting-based representation of PopNet-defined subpopulations that were depicted in panel **A**. Each row represents the genome of a single isolate, clustered into its respective subpopulations. Each column represents a 5,000 bp section. Section colors indicate the population to which that section shares ancestry.

Based upon *porB* typing and genome-wide phylogenetic analyses shown above, it is clear that the DGI phenotype is dispersed across the population and not a single clonal lineage. Together, these findings are consistent with a panmictic population structure for *Ngo* (17), but provide little insight into the relationship between DGI isolates. To effectively account for the impact of horizontal gene transfer (HGT) in rapidly evolving populations, we next took advantage of PopNet (18) to better understand population dynamics. PopNet divides the chromosome of each isolate into typically 5,000 base pairs in length and uses their pattern of SNPs to assess the similarities between isolates; this allows the identification of homologous regions across populations. The network visualization generated by this approach reveals relationships by color-coding each segment based upon the subpopulations that share it. Applying PopNet to the 360 isolates, we identified eight distinct subpopulations, assigned A–H. As evident in Fig. 4A, populations A (red), C (green), and F (yellow) cluster together, while the others are more distinct. The three central co-clustered subpopulations share patterns of variation across multiple regions of the chromosome, as indicated by their circular (Fig. 4A) or linear (Fig. 4C) representation of their genomes being interrupted by fragments of the other two colors. Isolates representing B, D, G, and H each cluster into their own populations. These are bridged to the other populations via edges (lines) that illustrate linkage to isolates from population A, perhaps indicating descendants of ancestors to these emergent subpopulations (Fig. 4A, inset). The relative lack of colored segments in subpopulations B (blue) and H (pink) reinforces the relative isolation of these two subpopulations since it indicates a lack of genetic exchange with other groups. Isolates from subpopulation E (orange) do not resolve edges to other populations at the threshold (>15% similarity) shown, indicating that it possesses the lowest genetic similarity to the other subpopulations, consistent with the fact that the genome maps are uniformly colored.

Subpopulations A and C both contain isolates associated with DGI (gray-filled centers). Despite their close evolutionary relationship with subpopulation F, all isolates in this latter group were non-invasive. On the contrary, despite its apparent genetic isolation, subpopulation E was found to be significantly enriched in DGI isolates relative to the overall population distribution (*P* = 0.04; Table 2). This distribution confirms that the DGI phenotype has emerged separately in multiple lineages and/or the genetic locus responsible for this phenotype has been transmitted between the populations. Interestingly, population E tends to have been isolated in the earlier years and consists primarily of samples from Manitoba (19 of 25 isolates; Fig. 4B), making it plausible to consider that these contributed genetic material that led to the emergence of the broader DGI outbreak in Canada. The DGI isolates exhibited a greater proportion of *porB1a* alleles than the non-DGI isolates, consistent with previous studies (8, 10, 12, 19). Conversely, subpopulations D, F, H, and I all lack DGI isolates and represent groups restricted to Ontario, largely isolated in 2015 and restricted to the *porB1b* allele. Beyond this, each subpopulation with DGI isolates (A, B, C, E, and G) includes isolates obtained over multiple years from several provinces, so they are not strictly distributed by region or date.

**TABLE 2:** Incidence of DGI isolates across PopNet-defined subpopulations^*a*^

Subpopulation	Total isolates	DGI isolates	*P*-value	Adjusted *P*-value	Odds ratio
A	98	38	0.09	0.19	0.67
B	63	17	0.25	0.31	0.7
C	61	7	0.11	0.19	1.99
**E**	**25**	**11**	**0.01**	**0.04**	**0.33**
G	9	1	0.69	0.69	2.07

^*a*^ This table presents a statistical analysis for the frequency of DGI isolates across PopNet-defined subpopulations that contain them. Significant enrichment for DGI isolates was performed using Fisher’s exact test; significant associations at an adjusted *P*-value of 0.05 are indicated in bold.

### Genetic associations among the DGI strains

To better focus on genetic elements with the potential association to the DGI phenotype, we examined patterns of shared ancestry across chromosomal segments (Fig. 4C). Consistent with the network figure (Fig. 4A), subpopulation E is largely homogeneous and indicates genetic isolation from other subpopulations. In contrast, subpopulations A, C, D, and F share many regions with common ancestry, with A and C sharing the greatest number of regions; this again reflects the network visualization and relatively frequent exchange of genetic material. Subpopulations D, F, and H, which did not contain any DGI isolates, possess few segments shared with A and C. Perhaps the most striking feature apparent is that subpopulation B has a high proportion of DGI isolates and yet shares relatively few segments with the others that contain DGI strains (A, C, and E); these shared regions appear interesting to explore for a potential contribution to the invasive potential.

We next sought to identify sequences that were unique to the invasive isolates. We identified 2,657 non-synonymous SNPs associated with the DGI phenotype (*P* < 0.20, Fisher’s exact test with Benjamini-Hochberg (BH) false discovery rate correction; Table S2). These variants occurred within 975 genes. Pathway enrichment analysis of the 2,657 variant SNPs revealed that they fell within 45 Gene Ontology categories (Fig. S3), including 12 associated with tRNA activity (e.g., methionyl-tRNA aminoacylation and ligase activity, tRNA pseudouridine synthesis). Also enriched were multiple pathways associated with the cell surface (e.g., polysaccharide biosynthesis, regulation of cell shape, cell wall organization, and lipid A biosynthesis), highlighting the potential variability of surfaces among strains and, possibly, between strains possessing DGI potential.

### Non-core genes associated with the DGI phenotype

The genomic analysis above only considered genes that were shared with the FA1090 reference strain. However, while FA1090 was originally isolated from a patient that had disseminated infection, the repeated passaging of the prototype strain might have resulted in the loss of certain genetic determinants. FA1090 also lacks several mobile elements commonly found in gonococcal strains, including the conjugative plasmid, β-lactamase plasmid, and the gonococcal genetic island (GGI), which could play a role in infection and dissemination. To account for this, we next examined whether any genes not encoded by FA1090 were enriched in the low-passage DGI isolates. For each isolate, we therefore used SPAdes (v.3.15 [20]) to assemble reads that did not align to the FA1090 genome into contiguous sequences (contigs), discarding any contigs with less than 3× coverage. From the 48,788 contigs generated from all isolates, GeneMark (v.1.14_1.25_lic [21]) annotated 91,596 putative genes. Of these, 38,379 were annotated to known orthologs through mapping to the EggNog database of orthologs (v.5.0 [22]). These comprised 5,431 unique orthologs, of which 68% were identified in only a single isolate. Focusing on the 259 unique orthologs present in at least five isolates, we identified 63 non-FA1090 genes that are enriched in DGI isolates relative to non-DGI isolates (*P* < 0.20, Fisher’s exact test with BH false discovery rate correction; Table 3). Notable among them are genes related to bacterial toxin/antitoxin systems (e.g., putative antitoxin of a bacterial toxin-antitoxin system YdaS/YdaT, and ParE toxin of the type II toxin-antitoxin system, *parDE*), as well as genes involved in conjugation and DNA transfer (e.g., TrbL/VirB6 plasmid conjugal transfer protein, bacterial conjugation TrbI-like protein, P-type DNA transfer ATPase VirB11). While the former may indicate some differential response to stress, the latter set of genes may suggest that DGI isolates of *Ngo* are more capable of participating in HGT.

**TABLE 3:** Non-FA1090 genes that were more likely to be found in DGI isolates than non-DGI isolates^*a*^

Ortholog	Adj. *P*-value	Odds ratio	Description
1S3G4	2.75E−06	5.80	Negative transcription factor for the prlF-yhaV operon
**2VRCS**	**1.62E−05**	**Inf**	**Bacterial protein of unknown function (DUF882)**
**2KR4G**	**3.89E−05**	**12.74**	**Bacterial DNA topoisomerases I ATP-binding domain**
COG0629	6.02E−05	12.52	Single-stranded DNA binding
2KT9J	6.57E−05	4.77	KilA-N
1NBP1	1.88E−04	3.31	PIN domain
2VX0H	1.88E−04	3.31	AAA ATPase domain
**1SDVS**	**3.71E−04**	**4.49**	**Lysozyme**
1GA2T	5.18E−04	4.22	prlF antitoxin for toxin YhaV toxin
1S1M6	5.18E−04	4.22	Protein of unknown function (DUF3577)
2VM6D	5.18E−04	4.22	TraE protein
2VPS2	5.18E−04	4.22	Domain of unknown function (DUF1845)
1N8IF	5.18E−04	4.15	Signal peptidase
1S3U8	5.18E−04	4.15	Required for disulfide bond formation in some periplasmic proteins.
1TKPF	5.18E−04	4.15	Type F conjugative transfer system protein TraW
2KVR0	5.18E−04	4.15	Putative helicase
**2VJKP**	**5.18E−04**	**4.15**	**Type F conjugative transfer system pilin assembly protein TrbC**
2VKJA	5.18E−04	4.15	PFAM TraU
**2VMSV**	**5.18E−04**	**4.15**	**F plasmid transfer operon protein**
2VU43	5.18E−04	4.15	Protein of unknown function (DUF1778)
2VU6M	5.18E−04	4.15	Lytic transglycosylase
**2VHE5**	**5.18E−04**	**4.09**	**Cobyrinic acid a,c-diamide synthase**
2VJ33	5.18E−04	4.09	TraG-like protein, N-terminal region
2VJFK	5.18E−04	4.09	Protein of unknown function (DUF3275)
**2VMWY**	**5.18E−04**	**4.09**	**Bacterial conjugation TrbI-like protein**
**2VNCU**	**5.18E−04**	**4.09**	**Type IV conjugative transfer system mating pair stabilization**
2VRNY	5.18E−04	4.09	Type IV conjugative transfer system lipoprotein (TraV)
2W4R9	5.18E−04	4.09	TraK protein
2VHPD	5.18E−04	4.03	Type IV secretion system protein TraC
**2VHPM**	**5.18E−04**	**4.03**	**Superfamily II DNA/RNA helicases, SNF2 family**
**2VQE5**	**5.18E−04**	**4.03**	**Type IV conjugative transfer system protein TraH**
2VV1R	5.18E−04	4.03	ParB-like nuclease domain
**2KUXP**	**1.25E−03**	**3.54**	**TraM recognition site of TraD and TraG**
2WDYM	1.68E−03	4.84	Putative antitoxin of bacterial toxin-antitoxin system, YdaS/YdaT
**1YAE4**	**3.75E−03**	**3.07**	**Acetyltransferase**
2KRT9	6.30E−03	3.83	KilA-N
1SSP7	1.14E−02	2.27	PIN domain
2K9NP	1.20E−02	4.78	Transglycosylase SLT domain
1S4ZS	3.26E−02	16.11	Conjugal transfer protein
2KQ7K	3.31E−02	3.38	Site-specific DNA-methyltransferase (adenine-specific)
2VT2C	3.31E−02	3.38	Restriction modification system DNA specificity domain
COG3617	5.86E−02	2.04	BRO family, N-terminal domain
**2VK4S**	**1.13E−01**	**1.92**	**DNA restriction-modification system**
2VVNE	1.40E−01	1.86	Pfam Zeta toxin
2KS6V	1.40E−01	2.00	ParE toxin of type II toxin-antitoxin system, parDE
1SN0B	1.40E−01	2.21	AAA ATPase domain
4HBP1	1.40E−01	2.21	Adenine-specific DNA methylase Mod
2KQGA	1.40E−01	1.94	VirB8 protein
2KQMF	1.40E−01	1.94	Type IV secretory system conjugative DNA transfer
2KQXH	1.40E−01	1.94	Conjugal transfer protein TraL
2KR9A	1.40E−01	1.94	Transglycosylase SLT domain
2KRRY	1.40E−01	1.94	Bacterial mobilization protein (MobC)
2KTK9	1.40E−01	1.94	Helix-turn-helix domain
2VR1H	1.40E−01	1.94	Transfer protein
2WI1R	1.40E−01	1.94	Type IV secretion system proteins
2KPHH	1.82E−01	1.89	Phage integrase family
2KPJ9	1.82E−01	1.89	P-type DNA transfer ATPase VirB11
2KQ4D	1.82E−01	1.89	Relaxase
2KQCJ	1.82E−01	1.89	Primase C-terminal 2 (PriCT-2)
2KQI5	1.82E−01	1.89	Bacterial conjugation TrbI-like protein
2KQK7	1.82E−01	1.89	CagE, TrbE, VirB family, component of type IV transporter system
2KR99	1.82E−01	1.89	TrbL/VirB6 plasmid conjugal transfer protein
2KRP6	1.82E−01	1.89	Type IV secretory pathway, VirB3-like protein
2KS3C	1.93E−01	Inf	Family of unknown function (DUF5397)

^*a*^ ID refers to the ortholog identification number from the EggNog 5.0 database. Bolded genes are present in the gonococcal genomic island. Tests for association were performed using Fisher’s exact test with Benjamini-Hochberg false discovery rate correction.

Among the set of DGI-enriched genes, and quite provocatively, the highest odds ratios are associated with two bacterial proteins (orthologs 2VRCS and 2KS3C) of uncharacterized function (DUF882, which has a predicted carboxypeptidase domain, and DUF5397); four other DGI-associated proteins are also undescribed (orthologs 1S1M6, 2VPS2, 2VU43, and 2VJKF) (Table 3). Beyond this, two of the top 3 most significant proteins are related to nuclease function, including a transcription factor proposed to negatively regulate the type II toxin-antitoxin system comprised of PrlF and the YhaV translation-dependent ribonuclease (ortholog 1S3G4; the PrlF antitoxin [1GA2T] was also highly associated) (23) and a type I topoisomerase (ortholog 2KR4G). We also sought to determine whether the expression of the phase-variable LgtA might be associated with DGI isolates, as has been previously suggested (24). We predicted the expression of LgtA through analysis of its consensus repeat lengths and found no significant association with expression and DGI status (*P* = 1, Fisher’s exact test).

**Fig 5:**
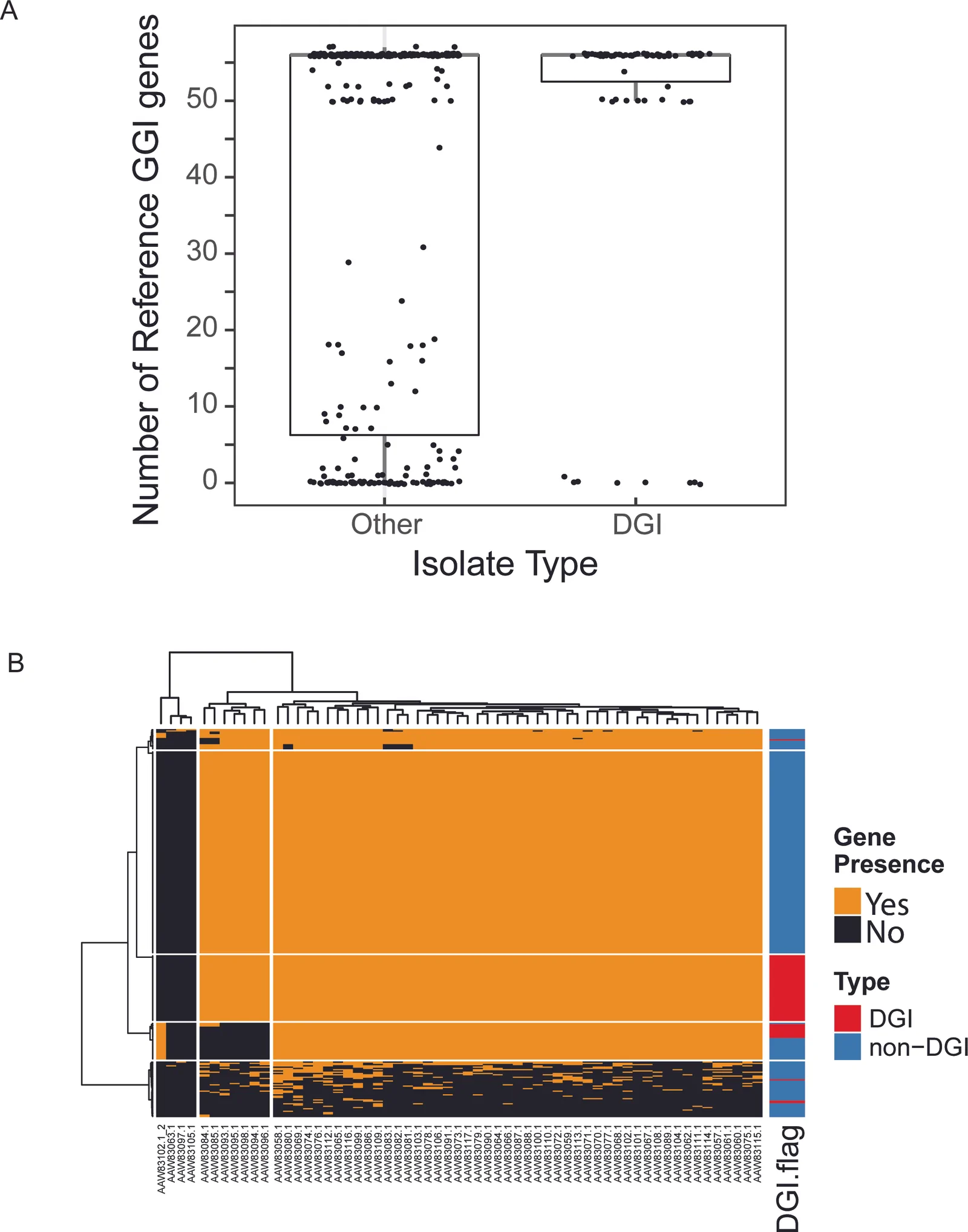
Distribution of GGI-associated genes associated with DGI phenotype. (**A**) Number of GGI genes in DGI and non-DGI *N. gonorrhoeae* isolates. (**B**) Presence and absence of 59 gonococcal genetic island genes across *N. gonorrhoeae* isolates.

A large number of DGI-enriched genes were related to a 57 kb GGI, which contains up to 63 genes. Not all strains express the GGI, which encodes a T4SS responsible for the extracellular release of linear single-stranded DNA (25). Since our reference strain (FA1090) does not encode it, we applied sequence similarity searches to the GGI of the *Ngo* strain MS11 to identify GGI genes in each isolate. DGI isolates were more likely to possess the full complement of GGI genes than non-DGI isolates (Fig. 5A, *P*-value = 2.96e−05, Welsh’s *t*-test). Our statistical analyses did not associate any single GGI gene with the DGI phenotype. However, a subsequent cluster analysis of the 59 GGI genes across the 301 isolates containing at least one GGI gene revealed a core set of 49 genes present in most (250 of 301) isolates (Fig. 5B), with an additional set of 7 genes found in 221 of these isolates.

## DISCUSSION

Amidst the rise of antibiotic resistance and a lack of gonococcal-specific vaccines, gonococcal infections represent a pervasive threat to global health. While invasive infections are infrequent, the recent emergence of clusters of DGI is concerning. DGIs have classically been more frequent in women, presumably because they are more likely to have asymptomatic infections that go untreated, yet the re-emergence of DGI has tended to include men and women in similar proportions (7–10). Immune suppression is a demonstrated risk factor (26, 27), yet this seems not to be a factor in most recent cases. While reduced access to healthcare during the COVID-19 pandemic was suggested as a plausible explanation for the increased incidence in California (9), the other incidences began prior to 2019 (7, 8, 10). The absence of obvious answers has frequently led to suggestions that the increased frequency in cases stems from the emergence of gonococcal isolates with an increased propensity for dissemination (5, 8–10). The difficulty in exploring this possibility stems from the routine diagnosis of gonococcal infection by nucleic acid amplification testing, which means isolates are not available. We took advantage of both the diverse gonococcal strain collection of the National Microbiology Laboratories of Canada and the routine isolation of *Ngo* from clinical cases in Ontario to select representative strains for phenotypic analysis and compare full genome sequences by complementary bioinformatic analyses of isolates from uncomplicated (mucosal) and disseminated gonococcal infections.

Besides the possibility of undiscovered/unmeasured epidemiologic or ecologic characteristics, the potential to cause invasive infection has classically, and intuitively, been linked to gonococcal resistance to complement-dependent killing, which occurs because the bacteria are able to sequester human-derived complement regulatory factors to their surface (12, 28). As with prior studies, most isolates from localized mucosal infections encode *porB1b*, while 74% of DGI isolates encode a *porB1a* allele of the PorB porin, which has been linked to complement regulatory factor H and C4BP binding (12). However, the exceptions in this simple relationship indicate that this alone cannot dictate the invasive phenotype.

It is also noteworthy that, because these isolates were obtained from national and provincial microbiology laboratories, we were unable to access detailed clinical markers such as polymorphonuclear leukocyte counts. As a result, it is possible that strains eliciting less overt local inflammation may be less likely to receive treatment, thereby extending the window of opportunity for dissemination.

To extend upon standard typing schemes, we considered whether binding to one or more complement components was a characteristic of the invasive strains, performing an exhaustive flow cytometry-based analysis of complement factor binding after exposing the bacteria to normal human serum. All isolates showed some association with FH and/or C4BP to varying degrees, yet this binding did not strictly correlate with that of C3 or the MAC, nor did it predict resistance to the bactericidal activity of serum. We observed that different isolates aggregate to different extents. While this phenotype did not relate to whether the strains were from invasive infection, we considered that the differential association with single versus aggregated bacteria might explain this difference. Indeed, there were some instances where FH and C4BP binding was markedly different between singlets and aggregates in a suspension of the bacteria; however, it is not evident how this could explain the marked serum sensitivity (15G0001 and 15G0003) or resistance (18G0016) of these cultures. We repeated the serum bactericidal assays with *Ngo* grown in the presence or absence of CMP-NANA, which confers serum resistance in some strains by virtue of their ability to sialylate their LOS (12); however, this did not reconcile the differences. Together, these factors indicate that the propensity to cause DGI is also facilitated by some other, as yet unrecognized, feature of the bacteria.

The availability of genome sequences generated from hundreds of isolates captured as part of enhanced public health surveillance allowed us to investigate the genetic determinants related to invasive infection. For this purpose, we included 360 isolates collected across a 7 year time frame (2013–2019) comprised of a broad spectrum of serotypes. When using a traditional NG-MAST analysis, five *porB* alleles were prevalent in the *Ngo* population. Of these, only *2206* and *5136* were significantly associated with DGI. The *2206* allele was spread across all included provinces, with 56 from Manitoba, 27 from Ontario, and 7 from Saskatchewan or Alberta, and has been prevalent since 2014. The proportion of DGI isolates from Manitoba and Ontario was comparable. Interestingly, all of the *5136* isolates were from Ontario and seem to have emerged after 2018, and the non-invasive isolates carrying this allele were derived from the eye (Table S1B). This makes it plausible to consider that a non-invasive *5136*-expressing isolate obtained some attribute(s) from the invasive *2206* lineage by horizontal genetic transfer.

In the context of the remarkable genetic plasticity of *Ngo*, even as a single strain is passaged in culture (29), we applied the population genomic tool, PopNet, to define population structure and identify loci exhibiting shared ancestry across isolates. Applying this tool revealed nine subpopulations, each representing a distinct lineage and separating isolates recovered from Manitoba from those obtained from Ontario, a segregation that was not evident with the standard SNP-based analysis. Moreover, the most recent Manitoba and Ontario DGI isolates (2018–2019) tended to be clustered together within subpopulation A. It is notable that certain subpopulations contain no DGI isolates because it suggests that the potential to cause invasive disease is not uniform among strains. However, with DGI isolates present among five of the nine subpopulations, this reinforces the possibility that the natural genetic competency of *Ngo* has allowed some DGI-associated genetic determinant(s) to be readily shared through genetic exchange.

Our PopNet-based genomic analyses identified 2,657 non-synonymous genomic variants across 975 genes that were enriched in the DGI isolates. The lack of strict correlation between any one allele and invasive disease is consistent with the phenotype being multifactorial. The analysis is complicated by the fact that mucosal isolates from uncomplicated infection may have the potential to cause DGI. Regardless, certain variants were significantly enriched in DGI isolates. These included three different non-synonymous mutations associated with the *purH* gene, which is involved in purine biosynthesis. The functional consequence of these changes is not evident based upon the variant sequences, yet *purH* and other genes upstream in the purine biosynthesis cascade were revealed by a genome-wide mutational screen to facilitate *Haemophilus influenzae* persistence during lung infection in mice (30), supporting the potential for a direct influence on gonococcal virulence. Also provocative, 13 of the DGI-enriched variants were associated with the cytosolic alginate O-acetyltransferase (NGO_0534, PacA), and 4 were within the associated periplasmic-localized PacB (NGO_0533), which modify peptidoglycan to confer resistance against hydrolysis by host lysozymes (31, 32). Classical studies have demonstrated that O-acetylated peptidoglycan from *Ngo* elicits a persistent arthritic response in rats, an effect substantially less apparent with non-acetylated peptidoglycan (33); this appears to relate to the persistence of undegraded acetylated fragments (34). Other significantly associated variants occurred in gene products involved in processes including cell envelope integrity, metabolism, and pilus assembly; however, 7 of the 25 most significant sequences encode hypothetical proteins (Table S2).

Neisseriae are naturally competent to a degree that the population structure is considered panmictic (35). Consequently, genetic determinants such as antimicrobial resistance or pathogenesis genes are naturally acquired (36, 37). Thus, it is feasible that the recent cluster of disseminated gonococcal isolates has acquired one or more genes not present in the FA1090 reference genome used to define our genetic variants, and would not then be evident from an alignment-based analysis. Indeed, we detected 63 genes that were enriched in DGI isolates but absent in FA1090 (Table 3). Six of these, including the two with the highest odds ratio, were proteins of unknown function, making them enticing to consider in future studies. Interestingly, four of the DGI-associated proteins are from toxin-antitoxin systems, which are often involved in the control of programmed cell death, stress tolerance, or plasmid maintenance (38). While *Ngo* FA1090 is predicted to encode two toxin-antitoxin systems (*vapBC* and *hicAB*), neither these nor those that we found to be enriched in the DGI isolates (*prlF*, *ydaS*, *zetA*, and *parE*) have previously been linked to pathogenesis. The GGI, a 57 kb genomic region with a relatively low G+C content, has previously been suggested to carry certain elements that are enriched in isolates from invasive infection (39). While we did find that the GGI was present in nearly all invasive isolates, we did not identify any individual gene that was significantly enriched in DGI isolates from those that have not been associated with invasive disease.

In this study, we took advantage of the routine collection of gonococcal isolates in Canada to compare uncomplicated and invasive infections. Phenotype analysis focused on bacterial aggregation, serum complement factor binding, and resistance to the bactericidal activity of serum, characteristics with clear potential to influence a disseminated outcome. While all isolates possessed the capacity to bind to the complement regulatory proteins, these together did not distinguish disseminated from non-disseminated *Ngo* isolates. A SNP-based genotype analysis of aligned sequences revealed no relationship between DGI isolates since these were scattered among those from uncomplicated disease across the phylogenetic tree. However, a PopNet-based analysis did differentiate Manitoba and Ontario DGI isolates and revealed that their association with invasive disease was not uniform since no cases were apparent within four of the nine subpopulations. This suggests that some feature of the bacteria must influence their capacity to disseminate; however, it is not clear if this is inherent in the subpopulations or has been horizontally transmitted from an invasive strain that has previously emerged. Based upon these analyses, it is enticing that there are a variety of non-synonymous genetic differences associated with the DGI phenotype, some of which were shared with the FA1090 but others which were shared among the invasive low-passage isolates but absent in this prototype strain. While our study provides a comprehensive genetic foundation for the re-emergence of DGI in Canada, we recognize that the path from genotype to invasive phenotype is likely governed by complex regulatory mechanisms. A rigorous evaluation of the transcriptional changes occurring when these strains encounter host-mimicking environments, such as human blood or serum, would offer critical insights into the immediate physiological adaptations required for systemic survival. Although the primary objective of this work was to identify stable, population-level genetic determinants evidenced by the 2,657 non-synonymous variants and 63 non-core genes enriched in DGI isolates, the high degree of donor-to-donor variability observed in phenotypic assays suggests that transient gene expression patterns also play a role. Consequently, future transcriptomic studies (RNA-seq) under host-mimicking conditions will be an essential next step to validate the functional impact of the DGI-enriched genetic variants identified here and to further delineate the mechanisms of gonococcal dissemination. Future studies must consider the potential for these to contribute to increased dissemination or, perhaps counterintuitively, reduced clinical manifestations during mucosal infection so as to promote the incidence of DGI by virtue of more infections being left untreated.

## MATERIALS AND METHODS

### Source of genome sequences and identification of genomic variants

The whole-genome sequences of 360 *Ngo* isolates obtained from PHO and NML were processed as described in Supplementary information (40), and genetic variants were identified using Genome Analysis Toolkit, filtered, and non-synonymous variants were further processed to identify their functional impact using SIFT 4G. Gene Ontology enrichment was performed to identify variants associated with DGI, using FA1090 as the reference. Phylogenetic trees were generated based on SNPs with a maximum likelihood algorithm. An optimal model was determined, and bootstrapping was performed for 100 iterations. A population network was constructed using PopNet (18), and chromosomal painting based on genome segment similarity was visualized using Cytoscape (v.3.8.2) (41).

For non-FA1090 genes, the non-aligned reads were assembled *de novo* into contigs, and putative genes were annotated against the eggNOG 5.0 database along with genes of FA1090 and used to filter putative genes matching FA1090 annotations. Genes corresponding to genes from five neisserial plasmid sequences—neisserial cryptic plasmid (NCBI ID: NC_001377 [42]); the prototypical β-lactamase plasmid (NCBI ID: NC_002098 [43]); and three others (NCBI IDs GU479464.1, GU479465, and GU479466 [44])—were similarly annotated. Contigs were aligned to genes associated with the GGI of *Ngo* strain MS11 (NCBI ID: AY803022.1) using BLAST+ (v.2.6.0 [45]); hits were found using GeneMark. The identity was determined using BLAST bitscore of over 80% alignment against *Ngo* prototype strain MS11 GGI. Differences in the number of GGI genes per isolate between DGI isolates and non-DGI isolates were tested using Welch’s *t*-test and plotted in a heatmap using ComplexHeatmap (46). Individual GGI genes were also tested for enrichment in DGI strains using Fisher’s exact test with a significance threshold of 0.20 and using the BH adjustment for multiple-testing correction. Only genes that were detected in at least five isolates were used for enrichment testing. We used PERF v.0.4.6 (47) to identify simple sequence repeats within LgtA for our collection of genomes.

### NG-MAST typing and porB allele comparisons

Sequences assembled *de novo* with SPAdes (v.3.15) were uploaded to pubMLST for automated NG-MAST typing (48). Sequences for *porB* alleles of interest were obtained from the pubMLST database, and AlphaFold (v.2.3.2) was used to predict protein structure. The Protein Data Bank Pairwise Structure Alignment tool (49) was used to align structures and determine structural differences between alleles. Allele sequences were aligned using ClustalW (50) and visualized with the R package msa v.1.37.3 using TEXshade implemented in the “msaprettyprint” function (51).

### Phenotypic assays

#### Serum resistance

*Ngo* strain survival was measured as percent CFU of the untreated strains. Briefly, 40 μL bacterial suspension was mixed with 60 μL of BHI + 10% IsoVitalex growth media, or HS, or heat-inactivated human serum. To examine the effect of sialylation on serum resistance, prepared cultures were pre-incubated in the presence or absence of a final concentration of 20 μM CMP-NANA at 37°C for 30 min prior to treating with HS (details provided in Supplemental information).

#### Assessment of complement deposition on *Ngo* strains

Samples were incubated at 37°C, 5% CO_2_ for either 15 (C3 and C5B-9) or 30 min (factor H and C4BP). Complement activation was stopped with ice-cold phosphate-buffered saline (PBS)/1% BSA/10 mM EGTA/10 mM MgCl_2_. Samples were washed twice with ice-cold 0.1% BSA/PBS and stained with a primary antibody for 45 min, washed twice with ice-cold 0.1% BSA/PBS and stained with a secondary antibody for 45 min, washed twice as described above, and fixed for 30 min with 4% paraformaldehyde solution in PBS. Samples were analyzed by flow cytometry (BD LSRII flow cytometer, Flow Cytometry Facility, UofT) on the same day. Data were analyzed and plotted using FlowJo v.10 (52). Antibody details and the analysis protocol are provided in Supplemental information.

## Data Availability

Sequence data for the isolates referenced in this paper have been deposited on the NCBI Sequence Read Archive under the following BioProjects: PRJNA1481706, PRJNA844269, PRJNA533242, and PRJNA516461. Individual accessions are listed in Table S1, containing isolate information. Additionally, assembled scaffolds have been uploaded to pubMLST under the *Neisseria* collection associated with this paper.

## AFTER EPUB

*[This article was published on 20 July 2026 with missing Author Contributions. The contributions were corrected in the current version, posted on 22 July 2026.]*

## SUPPLEMENTAL MATERIAL

Supplemental figures msphere.00051-26-s0001.pdf Fig. S1 to S3.

Supplemental information msphere.00051-26-s0002.docx Additional methodology details.

Supplemental tables msphere.00051-26-s0003.xlsx Tables S1 and S2.

ASM does not own the copyrights to Supplemental Material that may be linked to, or accessed through, an article. The authors have granted ASM a non-exclusive, world-wide license to publish the Supplemental Material files. Please contact the corresponding author directly for reuse.
