# Supplementary material for: Phenotypic diversity and shared genomic determinants among isolates causing a large incidence of disseminated gonococcal infections in Canada
Source: mSphere. 2026 Jul 20;11(8):e00051-26. doi: 10.1128/msphere.00051-26 (PMC13508793; doi:10.1128/msphere.00051-26)
Supplement: Supplementary file 1 [file msphere.00051-26-s0001.pdf]

Supplementary Figures:

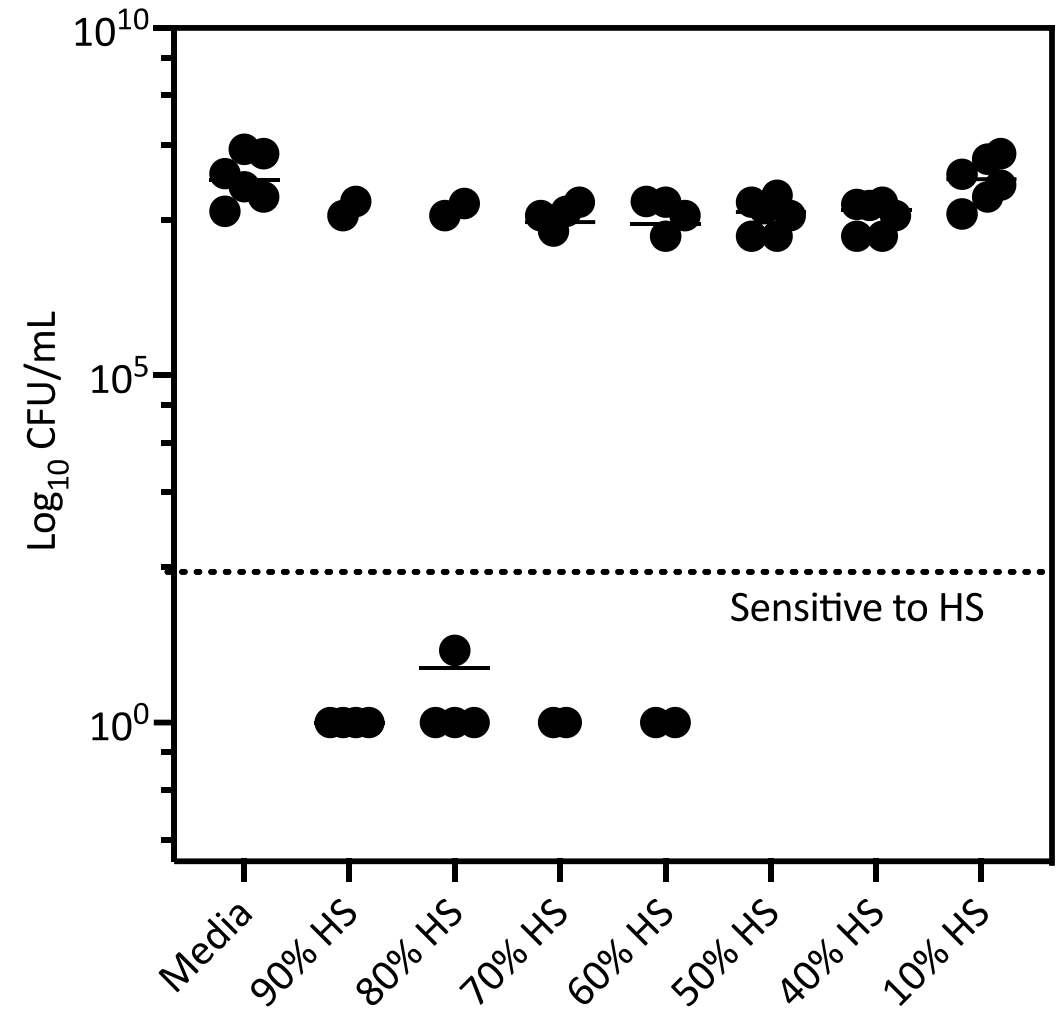

S. Fig. 1: Ngo isolates were randomly selected, and bacterial suspension was prepared in PBS with Mg^{2+} and Ca^{2+} to match the absorbance at 600nm~ 0.4, pelleted at 3000rpm and the pellets were resuspended in treatment media as described: media (BHI with 1% IsoVitalex), different concentrations of human serum (10-90%) in BHI with 1% IsoVitalex. Cultures were incubated for 60 min, washed once in PBS with Mg^{2+} and Ca^{2+}, serially diluted and plated on GC agar supplemented with IsoVitalex overnight to enumerate for CFUs.

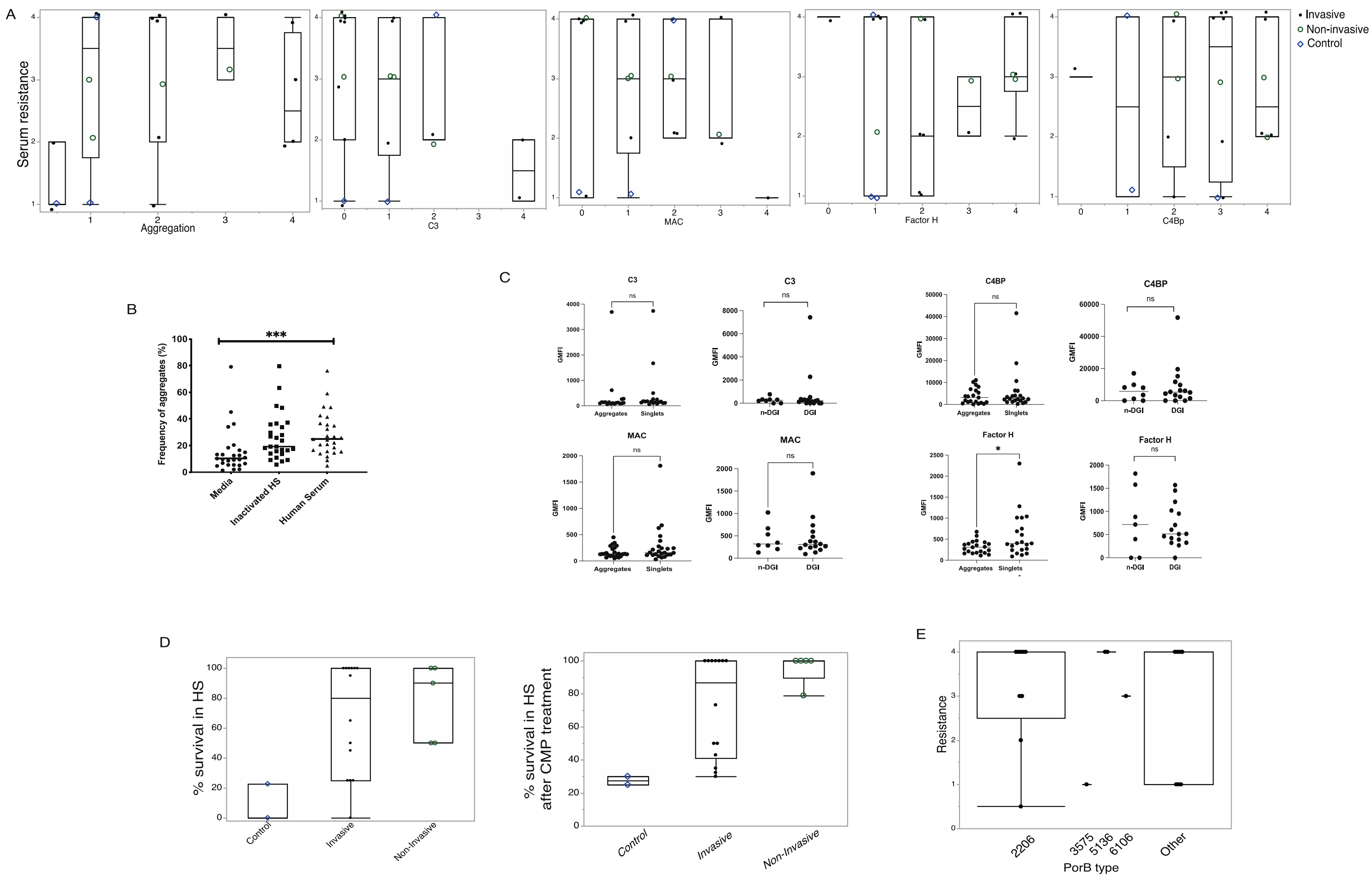

S. Fig. 2 : A) Correlation plot for serum resistance to aggregation (in the range of percentages where 1 (0-25%), 2 (26-50%), 3 (51-75%) and 4 (>76%), C3 deposition, MAC formation Fh and C4Bp recruitment represented as range of Geometric Mean Fluorescence Intensities (GMFI) of 1 (0-250), 2 (251-500), 3 (501-1000) and 4 (>1000). B) Effect of HS or heat inactived HS on aggregation. C) Comparison between GMFI of various complement factors based on aggregation phenotype or infection type. D) Percentage HS resistance of strains pre-treated with/without CMP-NANA as measured by CFUs. E) Effect of porB allele types on resistance of *Ngo* subset strains against active human serum.

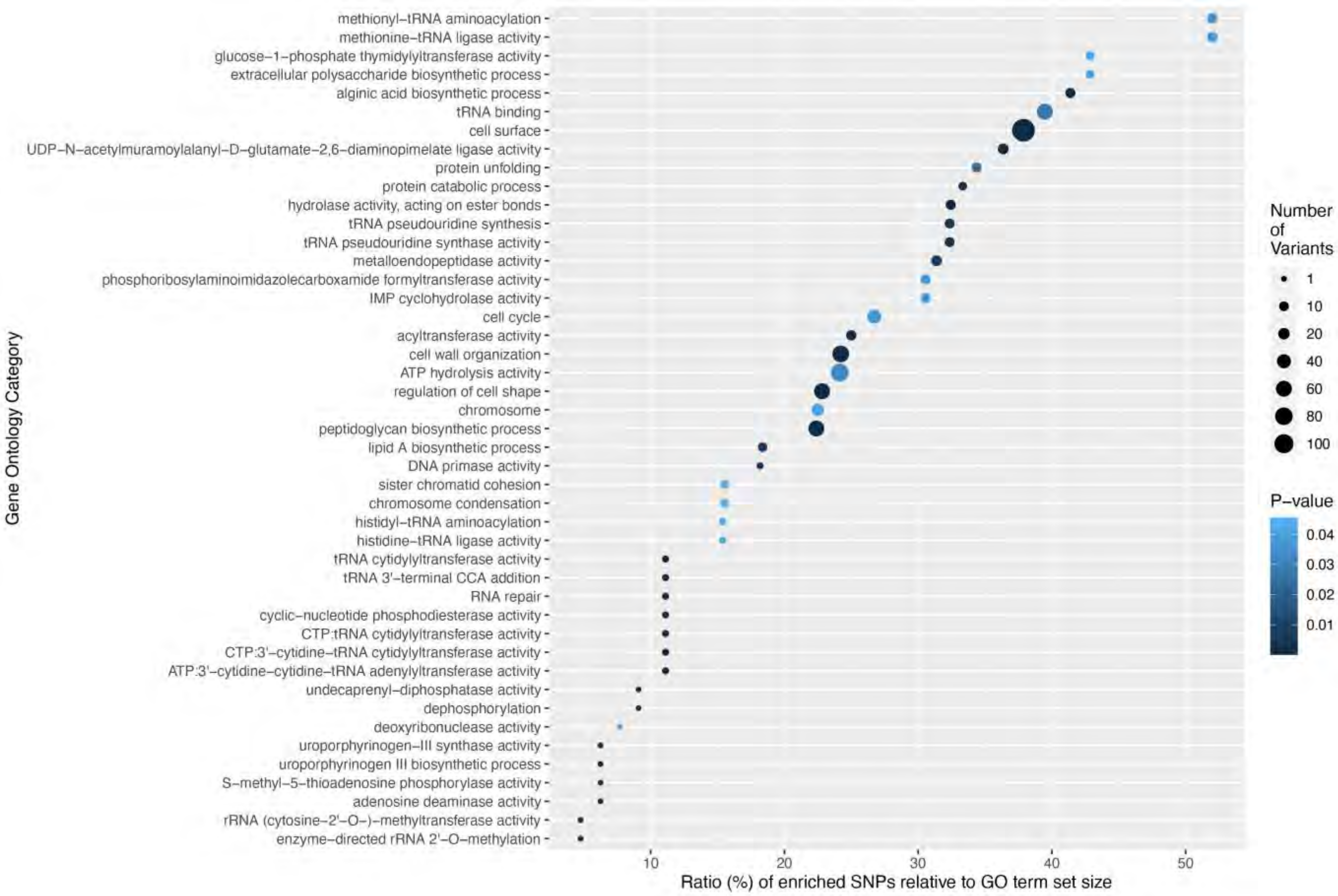

S. Fig. 3 Functional enrichment of SNPs relative to GO term set size. Size of the circle represents number of variants in each category and the color represents significance (p-value).
