# Supplementary material for: Phenotypic diversity and shared genomic determinants among isolates causing a large incidence of disseminated gonococcal infections in Canada
Source: mSphere. 2026 Jul 20;11(8):e00051-26. doi: 10.1128/msphere.00051-26 (PMC13508793; doi:10.1128/msphere.00051-26)
Supplement: Supplementary file 2 [file msphere.00051-26-s0002.docx]

**Supplementary information:**

Source of genome sequences and identification of genomic variants

Whole genome sequence data of 360 *Ngo* isolates (available sequences of strains from years 2013-19) were acquired from Public Health Ontario (PHO; Ontario, Canada) and the National Microbiology Laboratory (NML; Manitoba, Canada; **Supplemental Table 1**). Of these, 229 isolates are available at the sequence read archive of the National Center for Biotechnology Information at BioProject IDs PRJNA516461 (27 isolates) and PRJNA533242 (202 isolates). The remaining 131 isolates were acquired from PHO and NML directly.

To identify genetic variants (SNPs, insertions, and deletions), low quality reads were first filtered and trimmed using fastp (v.0.20.1; [40]) with the non-default parameters: qualified_quality_phred = 15; unqualified_percent_limit = 40; cut_right_window_size = 5; cut_right_mean_qu80ality = 20. Reads were aligned to a reference *Ngo* genome (FA1090 strain, genbank ID: NC_002946.2) using BWA (v 0.7.17; [41] with default parameters. Genetic variants were identified using the Genome Analysis Toolkit (GATK) (v.4.1.2.0; [42]). In the absence of a set of high-confidence genetic variants for *Ngo*, base quality and variant quality score recalibrations were not performed. Resulting variants were filtered using the following parameters: QualityDepth < 10.0, FisherStrand > 10.0, StrandOddsRatio > 3.0, RMSMappingQuality < 50.0, MappingQualityRankSumTest < -5.0. Significantly enriched non-synonymous genomic variants in DGI isolates were identified using Fisher’s exact test, with p-values adjusted using Benjamini and Hochberg’s correction with a significance threshold of < 0.20. Functional impact of variants was estimated using SIFT 4G [43].

To determine if mutations across related genes were associated with DGI, a Gene Ontology [44] enrichment analysis of non-synonymous variants was performed. For this analysis, the GO categories of each gene of *Ngo* FA1090 was retrieved from the gene annotations on the UniProtKB database [45]. Each non-synonymous genomic variant was then assigned to its respective GO terms [46]. Variants were ranked according to their p-values from the Fisher’s Test described above, and this list was used for the enrichment analysis, which was performed using the R-package clusterProfiler (v.4.0.5; [47]). Here, p-values of enriched GO-terms were adjusted using the Benjamini and Hochberg (BH) procedure with a significance threshold of 0.05.

**Population analysis**

Phylogenetic tree reconstructions were generated from concatenated alignments of single nucleotide polymorphisms (SNPs) using the R-packages phangorn (v.2.11.1; [48]) and ape (v.5.6; [49]). Trees were constructed with the maximum-likelihood algorithm. The optimal model was determined using phangorn’s modelTest function, which resulted in the General Time-Reversible model [50]. Bootstrapping was performed using 100 iterations. Trees were plotted using ggtree (v.3.0.4; [51]).

A population network of the isolates was constructed using the same alignment of SNPs as input into the population analysis tool, PopNet [18]. In brief, using segment lengths of 5000 base pairs, individual sections of each isolate were clustered using the Markov clustering (MCL) algorithm (inflation parameter = 8; pre-inflation parameter = 19). A ‘global’ similarity matrix is then constructed based on the number of segments placed in the same cluster for each pair of isolates, which was clustered again using MCL (inflation parameter = 6; pre-inflation parameter = 2) to define global populations. Genome segments for each isolate are colored based on the population with which it shares most similarity to (chromosome painting). Networks were visualized using Cytoscape (v. 3.8.2) [52]. The similarity of each isolate to one another (defined as the ratio of shared genomic sections) were used to define edges of the graph. In order to reduce the amount of edges in the network and resolve clusters, edges representing under 15% similarity between isolates were removed.

**Identification of non-FA1090 genes associated with DGI**

We searched for non-FA1090 genes using the remaining sequence reads that did not align to the reference strain as part of the variant analysis pipeline. For each isolate, the non-aligned reads were assembled *de novo* into contigs using SPAdes (v.3.15; [20]), and putative genes on these contigs were identified using GeneMark (v.1.14_1.25_lic; [21]). Contigs with less than 2X coverage were removed, and putative genes were annotated using the eggNOG mapper [53] against the eggNOG 5.0 database. The ortholog, as defined by the eggNog mapper, was used to annotate the genes [54]. Where unaligned reads assembled into FA1090 reference genes, the annotated genes of FA1090 were annotated using the eggNog mapper and used to filter putative genes matching these annotations. Genes corresponding to genes from 5 neisserial plasmid sequences (Neisserial cryptic plasmid, (NCBI ID: NC_001377; [55]); the prototypical B-lactamase plasmid (NCBI ID: NC_002098; [56]); and three others: NCBI IDs: GU479464.1, GU479465, GU479466; [57]) were similarly annotated. These “non-FA1090” genes were tested for enrichment in DGI isolates using Fisher’s exact test (p<0.2) with BH adjustment for multiple testing.

To identify genes associated with the gonococcal genetic island (GGI), contigs were first aligned to genes associated with the GGI hosted by *Ngo* strain MS11 (NCBI ID: AY803022.1) using BLAST+ (v.2.6.0; [58]); any hits were marked as putative GGI contigs. After this, putative genes on GGI contigs were found using GeneMark. The identity of the putative genes were determined by aligning them against *Ngo* prototype strain MS11 GGI genes using BLAST. The top alignment (by bitscore) determined the identity of the putative gene. A putative gene required a hit with over 80% sequence similarity to be annotated. Differences in the number of GGI genes per isolate between DGI isolates and non-DGI isolates was tested using Welch’s T-test. The presence or absence of each GGI gene per isolate was plotted in a heatmap using ComplexHeatmap [59]. Individual GGI genes were also tested for enrichment in DGI strains using Fisher’s exact test with a significance threshold of 0.20 and using the BH adjustment for multiple testing correction. Only genes that were detected in at least five isolates were used for enrichment testing.

**NG -MAST typing or porB alleles and structural comparison**

Sequences assembled de novo with SPAdes (v.3.15) were uploaded to pubMLST for automated NG-MAST typing [60]. Sequences for *porB* alleles of interest were obtained from the pubMLST database and AlphaFold (v2.3.2) was used to predict protein structure. The Protein Data Bank (PDB) Pairwise Structure Alignment tool [61]was used to align structures and determine structural differences between alleles. Allele sequences were aligned using ClustalW [62] and visualized with the R package msa v1.37.3 using TEXshade implemented in the “msaprettyprint” function[63].

**Serum resistance of DGI and n-DGI isolates**

*Ngo* cultures were grown overnight on GC agar supplemented with 1% isoVitalex, harvested and resuspended in BHI + 1% isoVitalex (media). OD_600_ was adjusted photometrically to 0.4 (approx. 10^8^ CFU/ml). 40 μl bacterial suspension was mixed with 60 μl of media (M), or human serum (HS), or heat-inactivated human serum (HI). To examine the effect of sialylation on serum resistance, prepared cultures were pre-incubated or not with final concentration of 20 μM CMP-*N*-acetylneuraminic acid (CMP-NANA) at 37^o^C for 30 mins prior to treating with HS.

**Flow cytometry to assess complement deposition on *Ngo* strains**

*Ngo* cultures were grown overnight on GC agar supplemented with 1% isoVitalex, harvested and resuspended in BHI + 1% isoVitalex (media). OD_600_ was adjusted photometrically to 0.4 (approx. 10^8^ CFU/ml). 40 μl bacterial suspension was mixed with 60 μl of media, or human serum, or heat-inactivated human serum. Samples were incubated at 37°C, 5% CO_2_ for either 15 (C3 and C5B-9 factors) or 30 minutes (Factor H and C4BP). Complement activation was stopped by adding 150 μl ice-cold phosphate-buffered saline (PBS)/1% BSA/10 mM EGTA/10 mM MgCl_2_. Samples were processed on ice, and pelleted at 500g, 4°C for 5 minutes and washed twice with ice-cold 0.1% BSA/PBS. The pellet was resuspended and stained with a primary antibody for 45 minutes, washed twice with ice-cold 0,1% BSA/PBS and stained with a secondary antibody for 45 minutes. The samples were washed twice as described above and fixed for 30 minutes with 200 μl of 4% paraformaldehyde solution in PBS. Samples were analyzed by flow cytometry (BD LSRII flow cytometer, Flow Cytometry Facility, UofT) on the same day. Data were analyzed and plotted using FlowJo v10 [64].

Primary and secondary antibodies for different complement factors were used as described: C3‑fragments were stained using primary poly-clonal goat-anti-C3 antibody at 1:1000 dilution and secondary donkey anti-goat IgG-Alexa 488 conjugate at 1:500 dilution. C5b-9 was stained using primary mouse anti-human-C5b-9 antibody clone ae11 at 1:1200 dilution and secondary goat anti-mouse Alexa 647 conjugate at 1:500 dilution. Factor H (FH) was stained using primary goat anti-human FH antibody at 1:1000 dilution and secondary donkey anti-goat Alexa 488 conjugate at 1:500 dilution. C4BP was stained using primary rabbit anti-human C4BP antibody at 1:500 dilution and goat anti-rabbit Alexa 647 conjugate at 1:500 dilution.

**Analysis of flow cytometry data**

To analyze bacterial populations in FlowJo v10, the main population in a FSC-A/SSC-A scatter plot was selected to exclude cellular debris with low FSC-A or SSC-A. Next, the main population in SSC-W/SSC-A was selected excluding scattering data at the upper and lower limits. In the next step of analysis two distinct populations of aggregates and singlets were defined in FSCH/FSC-A. The singlet population gate boundaries were based on population patterns observed in strains that showed no tendency of aggregation in fluorescence microscopy and transmission electron microscopy. The aggregate population gate boundaries were drawn around the cell populations larger than the singlet population. The same gates were used for all strains in all experiments.

Samples were analyzed for populational parameters regarding the percentage of singlets and aggregates under different experimental conditions, and the quantitative complement deposition in terms of fluorescence intensity. Histograms for aggregates and singlets were overlayed for the final representation using layout editor with Y axis manually adjusted to a maximum value of 500. Geometric mean fluorescence intensity (GMFI) was used to account for scattering data when quantifying complement deposition on samples. Due to strain-dependent variability, GMFI values were normalized to the negative control for each strain to allow better comparison of shifts between strains. For C3 and C5b-9 deposition, samples with heat-inactivated serum were selected as the negative control, since heat-inactivation abolishes complement activity of these factors. For FH and C4BP, samples in media, where these factors are absent, were used as the negative control since these complement factors are heat-stable and active in both heat-inactivated and fresh serum.

**Scanning electron microscopy of Ngo isolates**

Bacteria were streaked overnight on GC- agar supplemented with 1% IsoVitalex and incubated at 37^o^C for 16h. Bacteria was collected using cotton swabs and resuspended in PBS with Ca^2+^ and Mg^2+^, centrifuged at 3000 rpm for 5 minutes twice, and then resuspended in BHI supplemented with 1% IsoVitalex to an OD_600_ approximately 0.1 and grown in shaking incubator at 37^o^C until the OD_600_ reached 0.4 (typically 4-6 h). Bacteria were collected by centrifugation at 3000 rpm and resuspended in 2 ml phosphate buffer and fixed in equal volume of 3.7% formaldehyde overnight. The samples were then sent for further processing to the Microscopy Facility at the Faculty of Dentistry, University of Toronto. Samples were dehydrated through a series of ethanol rinses (30, 50, 70, 95 and 100%) and critical-point dried with liquid CO_2_. 3000x and 5000x images were processed for each strain sent for analysis.
